# Cholesterol Embolization Syndrome Post Invasive Arterial Procedure: A Case Report

**DOI:** 10.7759/cureus.46986

**Published:** 2023-10-13

**Authors:** Christeebella O Akpala, Grace Kim, David Froehling, Nneka Comfere, Sindhuja Sominidi Damodaran

**Affiliations:** 1 Dermatology, Mayo Clinic Alix School of Medicine, Rochester, USA; 2 Dermatology, Mayo Clinic, Rochester, USA; 3 Vascular Surgery, Mayo Clinic, Rochester, USA; 4 Dermatology/Pathology, Mayo Clinic, Rochester, USA

**Keywords:** cholesterol embolization syndrome, emboli, blue toes, purple toes, cabg, cholesterol emboli

## Abstract

Cholesterol embolization syndrome (CES) is a rare but systemic severe disease caused by the distal showering of cholesterol crystals after angiography, major surgery, thrombolysis, or anticoagulation. Here, we present a case of a 74-year-old male with a history of coronary artery disease, chronic kidney disease, peripheral vascular disease, antiphospholipid syndrome, and right internal carotid artery occlusion who developed purple discoloration and ulceration involving several toes two months after coronary artery bypass surgery. A broad differential diagnosis for blue toes was considered, and a biopsy was obtained, which revealed an arterial lumen filled with large cholesterol crystal spaces, confirming the diagnosis of CES. Treatment of CES remains a bimodal approach of supportive and prophylactic care. Although there is no direct evidence in favor of antiplatelet agents, their use seems reasonable because they have been shown to reduce the risk of other cardiovascular events in patients with extensive atherosclerosis. In this case, the patient's toe pain improved with the use of topical amitriptyline ketamine and has achieved complete resolution of pain and skin discoloration at a seven-month follow-up.

## Introduction

Cholesterol embolization syndrome (CES) is a multi-system embolic phenomenon that occurs when cholesterol and debris from atheromatous plaques of large arteries are released into the circulation. The emboli cause occlusion and inflammation of the small arteries and arterioles resulting in end-organ damage. Commonly affected organs are the skin, kidneys, brain, eyes, and intestines [1,2]. We report a case of CES presenting as purple discoloration and ulceration of toes with acute kidney injury two months after an invasive cardiac procedure.

## Case presentation

**Figure 1:**
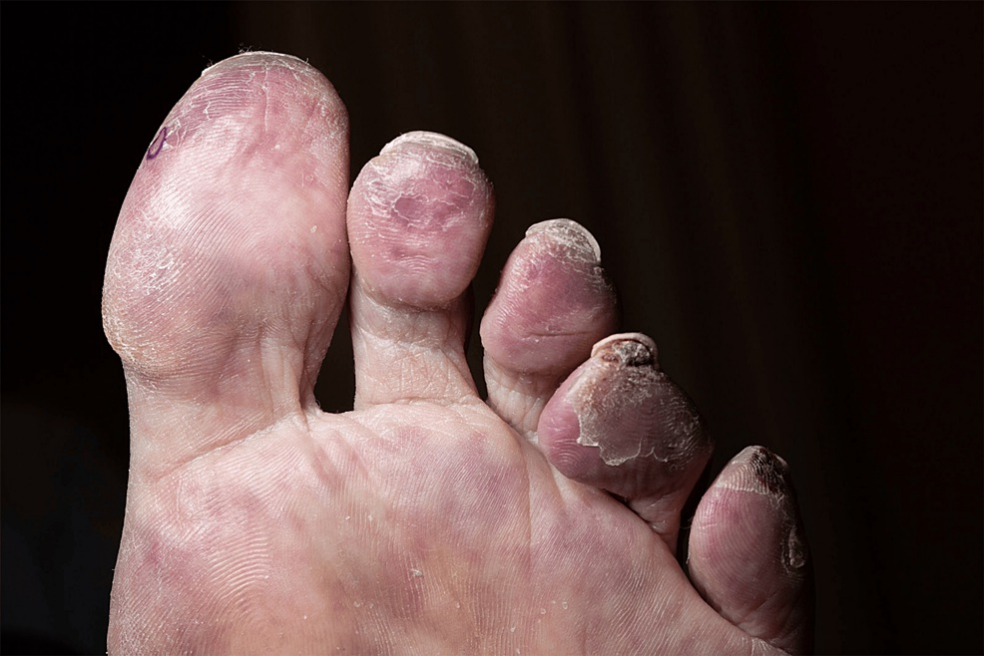
Clinical presentation of the patient with purple discoloration of toes and skin ulceration, subsequently identified as cholesterol embolization syndrome.

**Figure 2:**
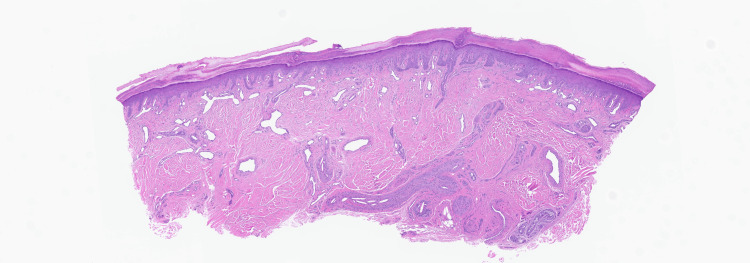
Blood vessel filled with cholesterol spaces in the deep dermis (x50).

**Figure 3:**
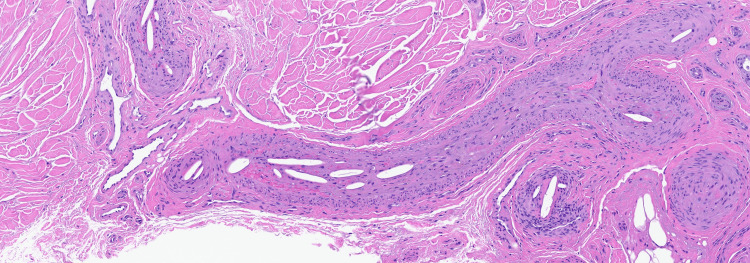
Arterial lumen filled with large cholesterol crystal spaces (x190).

A 74-year-old male presented to our clinic for evaluation of purple discoloration of the toes with an associated burning sensation of two months duration. His medical history included a recent four-vessel bypass procedure for severe coronary artery disease, chronic kidney disease, peripheral vascular disease, carotid artery occlusion, and hyperlipidemia. Approximately 10 weeks after the coronary bypass procedure, he started experiencing painful blue discoloration of his toes, which slowly worsened with time. Prior to our evaluation, he had a CT angiogram of the abdomen, pelvis, and lower extremities, which showed diffuse atherosclerotic disease and multiple areas of stenosis in the lower extremities, for which he was started on oral rivaroxaban. On exam, tender purple discolored patches and papules with ulceration involving several toes were seen (Figure 1). A broad differential was considered, including occlusive vasculopathy, vasculitis, cholesterol embolization, Raynaud's phenomenon, perniosis, and the antiphospholipid antibody syndrome. Laboratory workup showed an anti-nuclear antibody (ANA) level of 2.9 (reference range: <=1), elevated anti-centromere antibodies, antiphospholipid antibody IgM, anti-beta 2 glycoprotein antibody, and an elevated creatinine consistent with acute kidney injury. Cryoglobulin, cryofibrinogen, cold agglutinin, complements, monoclonal gammopathy screen, and anti-neutrophil cytoplasmic antibody were normal. Skin biopsy revealed characteristic cholesterol clefts within arterial lumina consistent with cholesterol emboli (Figures 2, 3). Direct immunofluorescence was negative. The patient was diagnosed with CES with associated anti-phospholipid antibody syndrome. He was started on enoxaparin and later transitioned to warfarin. His prior statin was continued, and he was started on 10 mg of ezetimibe, a selective cholesterol absorption inhibitor shown to reduce plaque formation. The patient's toe pain improved with topical amitriptyline 2% and ketamine 0.5% cream. At seven months follow-up, there was complete resolution of discoloration and pain in his toes, and renal function returned to baseline.

## Discussion

CES is a potentially life-threatening multi-system syndrome that usually occurs after invasive arterial or cardiac procedures, but can happen spontaneously or following anticoagulation therapy [1,2]. Predisposing risk factors for CES include atherosclerosis, male gender, advanced age, and other cardiovascular risk factors like hypertension, hyperlipidemia, smoking, diabetes mellitus, and peripheral vascular disease [3,4]. In the initial stages of CES, tissue damage is caused by mechanical occlusion of the small and medium-sized arteries resulting in ischemia or infarction of the affected organ. Subsequently, the presence of cholesterol crystals in the arterial lumen triggers a foreign body inflammatory reaction resulting in complement activation, leucocyte infiltration, and oxidative damage resulting in additional damage, which is followed by endothelial damage and eventual fibrosis [1,5].

The clinical presentation of CES depends on the underlying organ involvement. Patients can present with constitutional symptoms, acute kidney injury, elevated liver enzymes, infarction of the bowels, splenic infarction, stroke, and cutaneous involvement [2]. The skin is one of the commonly involved organs in CES with a myriad of presentations, including livedo reticularis, blue toes, ulceration, nodules, purpura, or gangrene [2]. CES lacks specific clinical or laboratory findings, and as such requires a tissue sample for a definite diagnosis. The presence of cholesterol clefts within the arterial lumen is a characteristic finding in CES. The cholesterol crystals are washed away with regular tissue processing and appear as biconvex needle-shaped empty spaces. When the specimen is processed using liquid nitrogen, the cholesterol crystals are preserved showing birefringence under polarized light [2].

There are no definite guidelines available for the treatment of CES. Treatment is primarily supportive care tailored toward the affected organ. Maintaining hemodynamic stability, pain control, and wound care are crucial in the acute phase. Hemodialysis may be needed for renal failure. Statins are usually considered because of their anti-inflammatory, lipid-lowering, and plaque-stabilizing properties, but the use of other treatments, such as anti-platelets, anticoagulants, and corticosteroids, in CES is controversial [1,6]. Anticoagulants such as warfarin are usually avoided, as there is a concern for increased risk for CES unless there is an underlying condition that requires its use [3]. Our patient received warfarin for an associated antiphospholipid antibody syndrome.

## Conclusions

In conclusion, the diagnosis of CES requires a high index of suspicion, particularly in patients with a history of atherosclerotic disease and or invasive procedures. Clinicians should be aware of the potential complications of this condition and provide appropriate supportive care to manage the symptoms and prevent further end-organ damage.
